# Atmospheric Cold Plasma-Assisted Egg White Protein-Casein Dual Complexation: Insights into Effects on Structural and Functional Properties

**DOI:** 10.3390/foods15213878

**Published:** 2026-10-29

**Authors:** Mohamed Salama, Rulin Jin, Chuhuan Hu, Shuangjian Li, Sameh S. El-Hadad, Tamer M. El-Messery

**Affiliations:** 1 Department of Food Science and Engineering, School of Agriculture and Biology, Shanghai Jiao Tong University, Shanghai 200240, China; rljin@sjtu.edu.cn (R.J.); chuhuanhu@sjtu.edu.cn (C.H.); shuangjianli@sjtu.edu.cn (S.L.); 2 Dairy Department, Food Industries and Nutrition Research Institute, National Research Centre, Dokki, Cairo 12622, Egypt; samehsobhygalal@yahoo.com; 3 Department of Food Science and Human Nutrition, College of Agriculture and Food, Qassim University, Buraydah 51452, Saudi Arabia

**Keywords:** cold plasma, egg white protein, casein, structural properties, protein–protein interaction

## Abstract

Atmospheric cold plasma (ACP) offers a promising approach for tailoring protein interactions through controlled oxidative modification, but its effect on mixed egg white protein (EWP) and dairy casein (CN) systems remains unclear. Hence, this study investigated the physicochemical, structural, and functional properties of EWP-CN dual systems (2:1) treated with ACP at 70 kV for 0–5 min, followed by heating at 90 ± 2 °C for 30 min. The results indicated that moderate ACP exposure (0.5–2 min) reduced particle size and turbidity, maintained solubility, and induced conformational rearrangement. Surface hydrophobicity increased from 535.16 ± 2.95 in the 0 min control to 718.60 ± 4.15 after 2 min of ACP treatment, representing a 34.3% increase and indicating partial unfolding and exposure of nonpolar regions. Over the same treatment interval, the carbonyl content increased significantly from 1.85 ± 0.10 to 3.09 ± 0.19 μmol carbonyl/g protein. When treatment was extended to 3–5 min, the carbonyl content increased nonlinearly, with the sharpest rise occurring between 2 and 3 min, reaching 6.02 ± 0.34 μmol/g at 5 min, approximately 3.25-fold higher than the control. These changes were accompanied by increased turbidity and particle size, as well as decreased solubility after heating, resulting in the formation of a visible gel after 5 min of treatment. Additionally, ACP treatment for 1 min improved emulsion activity and foaming performance, but emulsion stability remained below that of the control at all treatment times. Under the tested conditions, ACP treatment for 1–2 min represented a favorable modification window for balancing structural flexibility, colloidal stability, and functionality in EWP-CN dual systems. Treatment for 1 min generally enhanced colloidal and initial interfacial properties, while 2 min achieved the highest surface hydrophobicity. However, further formulation optimization is required to achieve long-term emulsion stability.

## 1. Introduction

Food proteins provide nutrition and essential functions, including solubility, emulsification, foaming, gelation, and texture. These properties are governed by protein structure and intermolecular interactions, making structural regulation essential for developing stable, high-quality protein foods [1,2]. Among these, egg white proteins (EWPs) are attractive ingredients because of their high nutritional quality and versatile functional properties. EWPs contain several proteins with distinct molecular structures, particularly ovalbumin, ovotransferrin, ovomucoid, ovomucin, and lysozyme. Ovalbumin represents 54% of the total protein and contributes substantially to the gelling, foaming, and emulsifying behavior of egg whites [3]. Despite their advantages, EWPs are sensitive to temperature, pH, ionic strength, and concentration, which can induce unfolding and aggregation, reducing solubility and interfacial activity. During heating, proteins associate through hydrophobic, electrostatic, hydrogen bonding, and sulfhydryl–disulfide interactions. Limited unfolding improves adsorption and network formation, whereas extensive aggregation forms insoluble particles [4,5]. Herein, controlled modification is essential to enhance EWP performance in emulsified, aerated, gelled, and high-protein foods.

Combining proteins with complementary structures can overcome the limitations of individual ingredients. Casein (CN), the major bovine milk protein, comprises αs1-, αs2-, β-, and κ-CNs [6,7]. Unlike globular EWPs, CN has a flexible and disordered structure with exposed hydrophobic and charged regions. These features support its hydration, emulsifying, and stabilizing properties. Accordingly, CN can interact with partially unfolded EWPs through electrostatic interactions, hydrogen bonding, and hydrophobic interactions, regulating aggregation and interfacial adsorption to form a more stable dual-protein system [8]. Salama et al. [5] investigated the phase behavior and structural properties of EWP-CN mixtures and demonstrated that appropriately controlled complexation produced a stable colloidal system. Subsequent thermodynamic and microrheological analyses confirmed that CN influenced the molecular mobility, aggregation behavior, and thermal response of EWPs [8]. Nonetheless, increasing the total protein concentration of EWP-CN complexes by over 3.0% resulted in gelation behavior and aggregation when subjected to high heat treatment [9]. Therefore, a rapid physical process capable of regulating these complex interactions without extensive heating or chemical crosslinkers is needed.

Atmospheric cold plasma (ACP) has emerged as a promising non-thermal technology for the targeted modification of food proteins. ACP is generated by applying electrical energy to a gas at atmospheric pressure, producing energetic electrons, ions, excited molecules, ultraviolet photons, and reactive oxygen and nitrogen species [10]. Depending on the working gas and treatment environment, important reactive species can include ozone, hydroxyl radicals, hydrogen peroxide, superoxide, nitric oxide, nitrite, and nitrate. These species interact with amino acid side chains and alter disulfide bonds, hydrophobic domains, surface charge, and protein secondary and tertiary structures [11]. Bu et al. [12] demonstrated that individual plasma-generated species produced distinct structural and functional responses, highlighting the importance of plasma composition and treatment intensity. Nasiru et al. [13] reported that ACP altered the secondary structure of EWPs, decreased particle size, and improved solubility, foaming, emulsification, and water- and oil-holding properties. Likewise, Baek et al. [14] found that moderate ACP-induced oxidation increased the surface hydrophobicity and exposed the sulfhydryl content of EWPs, improving emulsion stability. However, prolonged treatment promoted aggregation and reduced the functional improvement. Concurrently, Zhang et al. [15] demonstrated that controlled ACP treatment modified the phosphorylation degree, oxidation status, conformation, and functional properties of sheep milk casein. These results indicate that CN can undergo plasma-induced molecular reorganization while retaining its capacity to stabilize food colloids.

Recent studies have examined ACP-treated EWPs and CN separately, leaving its effect on their co-complexation unclear. Their contrasting structures, charges, and oxidative susceptibilities may produce unpredictable responses from single-protein systems. This study therefore evaluated the time-dependent effects of ACP on the physicochemical, structural, colloidal, and functional properties of EWP-CN mixture systems and the transition from limited structural modification to extensive aggregation. The results provide evidence of ACP-dependent changes in the mixed protein system and support the development of physically modified EWP-CN ingredients.

## 2. Materials and Methods

### 2.1. Materials

Fresh hen eggs and soybean oil were obtained from a local supermarket. Casein (92% purity) was supplied by Shanghai Yuanye Bio-Technology Co., Ltd. (Shanghai, China). 8-Anilino-1-naphthalenesulfonic acid (ANS) and 2,4-dinitrophenylhydrazine (DNPH) were purchased from Sigma-Aldrich (St. Louis, MO, USA). Guanidine hydrochloride (GuHCl) was acquired from Shanghai Aladdin Biochemistry & Technology Co., Ltd. (Shanghai, China). All other chemicals were of analytical grade.

### 2.2. Preparation of EWPs

Egg whites were separated from infertile hen eggs and stirred at 500 rpm and 4 °C for 12 h using a magnetic stirrer (Model 85-2, Shanghai, China). Then, the samples were diluted with deionized water at a water-to-EWP ratio of 2:1 and equilibrated at 4 °C for 6 h. Insoluble ingredients were removed by filtering through a 1 mm mesh filter (Jinteng Experimental Equipment Co., Ltd., Tianjin, China). The filtrate was spray dried (Spray Dryer-1500, Shanghai, China) at an inlet air temperature of 180 °C and a feed rate of 600 mL/h. The collected powder was sealed in airtight containers and stored at −20 °C until further use. The protein content of the spray dried EWP powder was 72.0 ± 0.25% (*w*/*w*, dry basis), as determined by the Kjeldahl method [5].

### 2.3. Preparation for ACP-Assisted EWP-CN Dual Complexation

EWP powder was dispersed in deionized water to obtain a 4% (*w*/*v*) stock solution and stirred until fully hydrated. A 4% (*w*/*v*) CN solution was prepared in a similar manner, with the pH (FE20 pH meter, Mettler-Toledo, Shanghai, China) adjusted to 11.0 using 4 M NaOH to ensure complete dissolution. Powder quantities were corrected for the measured EWP content (72.0 ± 0.25%, dry basis) and casein purity (92%); thus, 4% refers to the actual protein content. Following Salama et al. [8], the EWP and CN solutions were mixed at a fixed 2:1 volume ratio, producing a final protein concentration of 4% (*v*/*v*). The mixture was adjusted to pH 7.0 and stirred at 400 rpm and 25 °C for 2 h. Aliquots (20 mL) were transferred to sterile Petri dishes and treated under ambient air conditions using a dielectric barrier discharge ACP system (Suzhou Opus Plasma Technol. Co., Suzhou, China). The system comprised high-voltage and grounded electrodes separated from the treatment zone by dielectric barriers, with an electrode gap of 50 mm and the sample surface positioned 10 mm below the high-voltage electrode. Based on the protocol of Abbas et al. [16], the applied voltage was set to 70 kV and the treatment durations were adjusted to 0.5, 1, 2, 3, and 5 min. The untreated sample was designated as the 0 min control. Following ACP treatment, each 20 mL sample was divided into two equal 10 mL aliquots. One aliquot was retained without heating, whereas the other was transferred to a glass vial and heated in a digital water bath (HH-4, Changzhou, China). The 30 min holding period began after the sample reached 90 ± 2 °C. Post-heating, the samples were immediately cooled in an ice bath to room temperature and then subjected to the designated analyses. Both unheated and heated samples were analyzed for colloidal stability, particle size, electrokinetic properties, UV absorption, and intrinsic fluorescence. Surface hydrophobicity, carbonyl content, and emulsifying and foaming properties were measured only prior to heating to isolate the impacts of ACP. In our previous work, EWPs, CN, and their mixtures were systematically compared and characterized [8]. Building on those findings, the present study was designed specifically to evaluate the effects of ACP treatment on the previously established EWP-CN (2:1) system rather than to reassess the individual protein fractions. A total protein concentration of 4% (*w*/*v*) was selected because our previous results showed that EWP-CN complexes containing more than 3% protein were prone to heat-induced aggregation and gelation [9]. In this study, the plasma-generated reactive oxygen and nitrogen species (RONS) were not directly characterized; therefore, the observed effects are interpreted as overall ACP-induced changes rather than effects of specific reactive species.

### 2.4. Turbidity and Solubility Measurements

Turbidity was determined following Salama et al. [9]. Samples were adjusted to 10 mg/mL and measured at 600 nm using a UV/Vis spectrophotometer (Thermo Fisher Scientific, Waltham, MA, USA), with distilled water as the blank (100% transmittance). Turbidity was calculated as 100 - transmittance. For solubility measurements, samples were centrifuged at 10,000× *g* and 4 °C for 15 min using a refrigerated centrifuge (Sigma 3-30K, Sigma Laborzentrifugen GmbH, Osterode am Harz, Germany). Protein contents in the supernatant (A) and original dispersion (B) were determined using the method of Lowry et al. [17]. Solubility was calculated using the following equation:

(1) $$\mathrm{Solubility}\;\left(\%\right)\;=A/B\;\times \;100$$

### 2.5. Particle Size and Zeta Potential Measurements

Particle characteristics and zeta potential were measured using a Zetasizer Nano ZS (Malvern Instruments Ltd., Worcestershire, UK). All samples were diluted with distilled water to 1 mg/mL to minimize multiple scattering and concentration effects. The Z-average and polydispersity index (PDI) were determined by dynamic light scattering in disposable polystyrene cuvettes at 25 °C. Zeta potential, electrophoretic mobility, and electrical conductivity were measured by electrophoretic light scattering using folded capillary cells (DTS1070) at 25 °C. All measurements were performed in triplicate [5].

### 2.6. UV Spectrum

UV absorption spectra were obtained following Li et al. [4] using a NanoDrop 2000C UV/Vis spectrophotometer (Thermo Fisher Scientific, Wilmington, DE, USA). Samples were diluted to 1 mg/mL, transferred to 1 cm quartz cuvettes, and scanned from 240 nm to 400 nm at 25 °C, with distilled water used as the blank sample.

### 2.7. Intrinsic Fluorescence Spectrum

Intrinsic fluorescence spectra were recorded using an F-4600 fluorescence spectrophotometer (Shimadzu, Tokyo, Japan) to assess changes in the aromatic amino acid residues’ microenvironment. Samples were diluted to 0.2 mg/mL and excited at 295 nm. Emission spectra were collected from 300 nm to 450 nm at 25 °C using 5 nm excitation and emission slits and a photomultiplier tube voltage of 400 V [4].

### 2.8. Surface Hydrophobicity Measurement

Surface hydrophobicity (H_0_) was determined using ANS according to the methodology of Salama et al. [9]. Samples were diluted with 10 mM phosphate buffer (pH 7.0) to 0.01–0.05% (*v*/*v*). Each dilution (4 mL) was mixed with 20 µL of 8 mM ANS, vortexed, and incubated in the dark for 15 min. Fluorescence intensity was measured using an F-4600 spectrophotometer at excitation and emission wavelengths of 390 nm and 470 nm, respectively. Both the excitation and the emission slit widths were set to 5 nm. H_0_ was calculated as the initial slope of fluorescence intensity versus protein concentration. All measurements were performed in triplicate.

### 2.9. Protein Oxidation Measurement

Protein oxidation was evaluated by determining carbonyl content according to Hu et al. [18] with modifications. Samples were diluted to 5 mg protein/mL, and 0.5 mL aliquots containing 2.5 mg protein were combined with 0.5 mL of 10 mM DNPH prepared in 2 M HCl. After incubation at 40 °C for 1 h in the dark, 3 mL of 20% trichloroacetic acid was added to terminate the reaction. The mixtures were centrifuged at 10,000× *g* and 4 °C for 15 min, and the collected pellets were washed three times with 1 mL of ethanol:ethyl acetate (1:1, *v*/*v*) to remove unreacted DNPH. The final pellets were then dissolved in 1 mL of 6 M GuHCl prepared in 20 mM potassium phosphate buffer (pH 6.5) and incubated at 37 °C for 15 min until completely dissolved. The absorbance was recorded at 370 nm using a UV/Vis spectrophotometer, with blanks prepared by replacing the DNPH solution with 2 M HCl. Carbonyl content was calculated using a molar extinction coefficient of 22,000 M^−1^ cm^−1^, normalized to the initial 2.5 mg of protein, and expressed as μmol carbonyl/g protein. All measurements were performed in triplicate.

### 2.10. Emulsifying Properties

The emulsifying activity index (EAI) and emulsion stability index (ESI) were determined according to Dong et al. [19]. Protein dispersion (30 mL) was combined with soybean oil (10 mL) and homogenized (XHF-DY, Ningbo, China) at 10,000 rpm for 2 min. At 0 and 30 min, 100 µL aliquots were collected from the bottom of the emulsion and diluted with 0.1% (*w*/*v*) SDS up to 5 mL. The absorbance was measured at 500 nm using a UV/Vis spectrophotometer. EAI and ESI were calculated using the following equations:

(2) $$\mathrm{EAI}\;\left(m^{2}/g\right)\;=\;(2\;\times \;T\;\times \;A_{0}\;\times \;N)/(C\;\times \;\phi \;\times \;(1\;-\;\phi )\;\times {\;10}^{4})$$

(3) $$\mathrm{ESI}\;\left(\min\right)={(A}_{0}\;\times \;30)/\left(A_{0}\;-{\;A}_{30}\right)$$

where $A_{0}$ and $A_{30}$ are the absorbance at 500 nm at 0 and 30 min, respectively. T is 2.303, N is the dilution factor, C is the protein concentration in the aqueous phase (g/mL), and ϕ is the oil volume fraction.

### 2.11. Foaming Properties

Foaming capacity (FC) and foam stability (FS) were evaluated using the method of Nasiru et al. [13] with some modifications. A 20 mL portion of the sample ($V_{L}$) was homogenized at 10,000 rpm for 2 min. The initial foam volume was recorded after 1 min ($V_{0}$), and the remaining foam volume was measured after standing at 25 °C for 30 min ($V_{T}$). FC and FS were calculated as follows:

(4) $$\mathrm{FC}\;\left(\%\right)\;={\;V}_{0}/V_{L}\;\times \;100$$

(5) $$\mathrm{FS}\;\left(\%\right)={\;V}_{T}/V_{0}\;\times \;100$$

### 2.12. Statistical Analysis

All measurements were performed in triplicate, and data are presented as mean ± standard deviation. Differences among treatments were assessed by one-way analysis of variance (ANOVA) using IBM SPSS Statistics 27.0 (IBM Corp., Armonk, NY, USA), with significance established at *p* < 0.05. Figures were generated using Origin 2026 (OriginLab Corporation, Northampton, MA, USA).

## 3. Results and Discussion

### 3.1. Colloidal Stability of ACP-Treated EWP-CN Dual System

**Figure 1:**
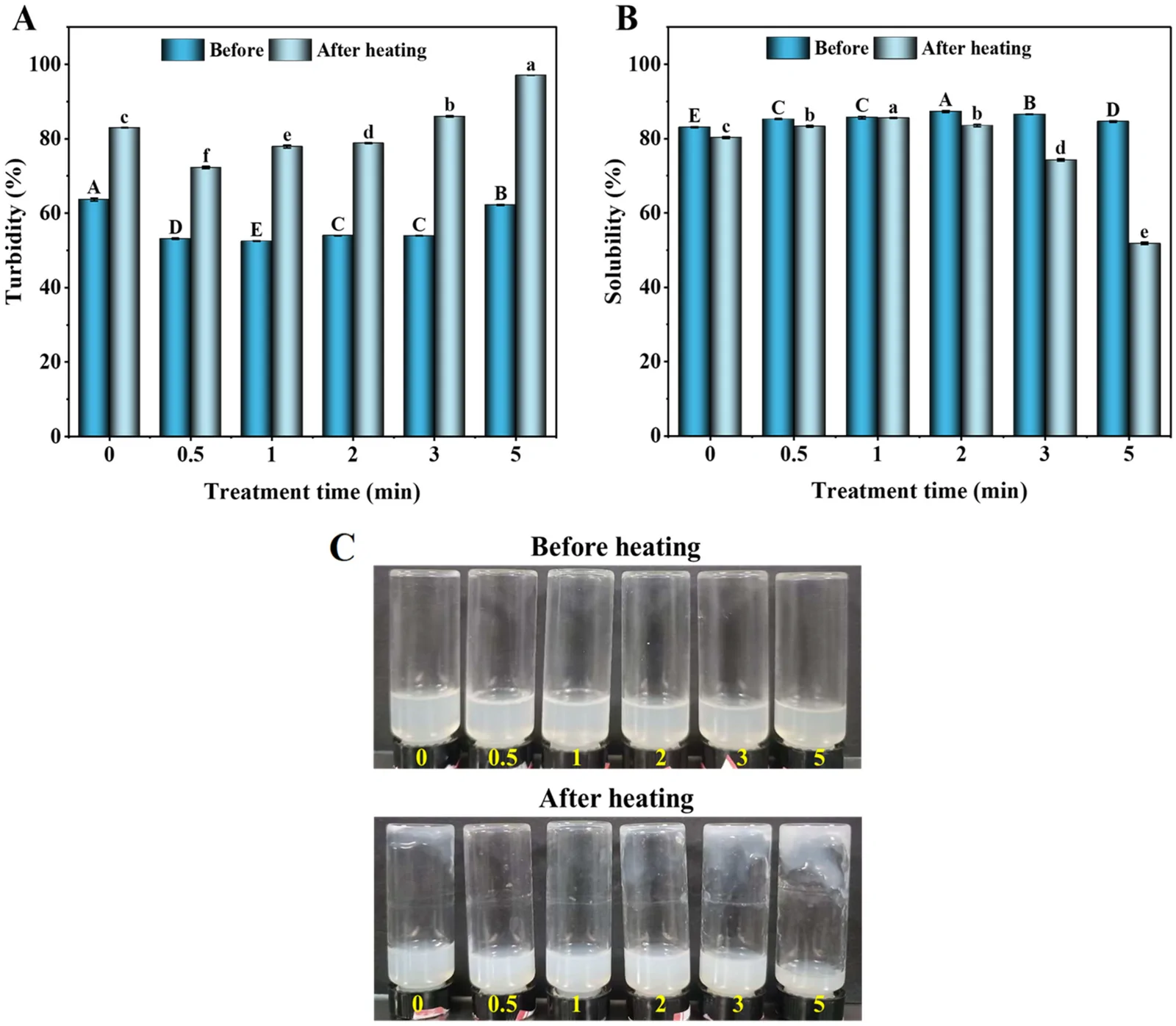
Turbidity (**A**); solubility (**B**); and visual appearance (**C**) of ACP-treated EWP-CN dual systems at different times (min) before and after heating at 90 ± 2 °C for 30 min. Different letters represent significant differences at *p* < 0.05, with capital letters denoting the treatments before heating and small letters referring to the same treatments after heating.

Turbidity and solubility are key indicators of protein colloidal stability because they reflect aggregate formation and the ability of proteins to remain dispersed in the aqueous phase [8]. To distinguish the two processing stages, changes in unheated samples were discussed as ACP-induced effects, while changes observed post-heating were interpreted as thermal aggregation or gelation modulated by ACP pretreatment. Figure 1 illustrates the effects of ACP for different time periods on the turbidity and solubility of EWP-CN complexes. Before heating, ACP treatments for 0.5, 1, 2, and 3 min significantly reduced turbidity from approximately 64% to 52–54%, while solubility increased from 83% to a maximum of 88% at 2 min of ACP treatment (*p* < 0.05). This indicated partial disaggregation and improved protein hydration, consistent with the smaller particles and increased solubility reported for ACP-treated EWPs by Nasiru et al. [13]. At 5 min of treatment, turbidity increased significantly and solubility declined slightly, suggesting the onset of protein reassociation. Heating clearly differentiated the ACP doses. Treatment for 0.5, 1, and 2 min maintained relatively low turbidity and high solubility, with 1 min providing the highest solubility after heating (86%). Conversely, the treatment for 3 min remarkably increased turbidity to 87% and reduced solubility to 73%, indicating intensified aggregation (*p* < 0.05). At 5 min, turbidity reached approximately 97%, solubility decreased to 52%, and a self-supporting gel formed, as confirmed by the visual appearance shown in Figure 1C.

Moderate ACP generated reactive species that partially unfold proteins, disrupt weak interactions, and expose charged and hydrophilic groups. The presence of flexible CN chains may have contributed to steric and electrostatic stabilization of the mixed protein system [8]. Nevertheless, prolonged ACP promoted excessive oxidation, hydrophobic exposure, and sulfhydryl-disulfide rearrangement [14]. Liu et al. [20] elucidated that controlled plasma treatment inhibited ovalbumin aggregation, while excessive exposure resulted in dose-dependent aggregation. Moreover, subsequent heating strengthened hydrophobic association and disulfide crosslinking, converting aggregated proteins into a continuous gel network. Overall, short ACP exposure improved EWP-CN dispersion and heat stability, whereas 5 min of treatment overprocessed the proteins and promoted heat-induced gelation.

### 3.2. Particle Size and Electrokinetic Analysis

**Figure 2:**
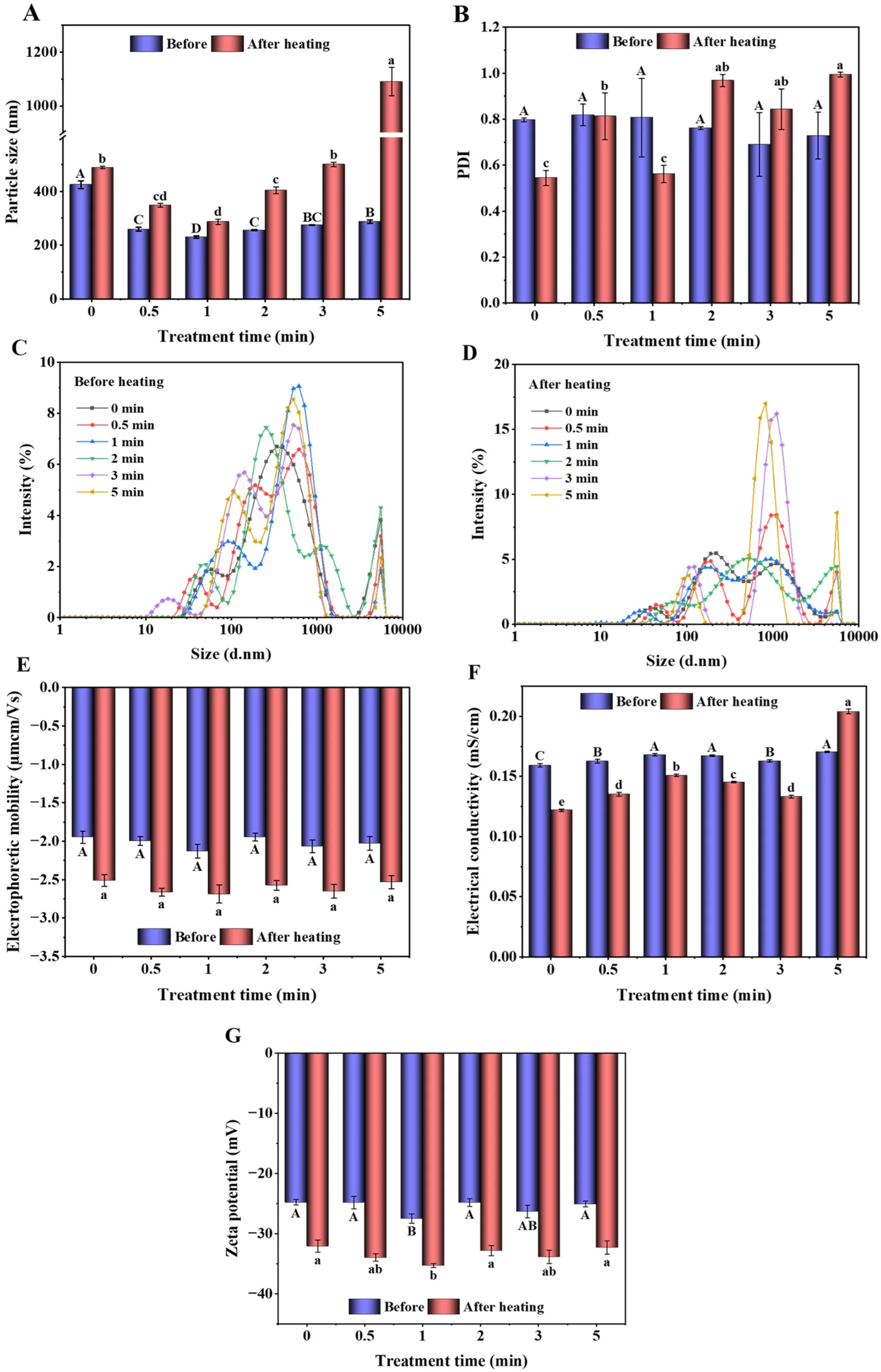
Changes in particle size (**A**); PDI (**B**); particle size distribution before (**C**); and particle size distribution after heating (**D**) of ACP-treated EWP-CN dual complexes at different times (min). Changes in electrophoretic mobility (**E**); electrical conductivity (**F**); and zeta potential (**G**) of ACP-treated EWP-CN systems at different times. Different letters represent significant differences at *p* < 0.05, with capital letters denoting the treatments prior to heating and small letters referring to the same treatments post-heating.

Particle size and surface charge are complementary indicators of protein colloidal stability, describing the extent, heterogeneity, and electrostatic regulation of aggregation. As depicted in Figure 2A, ACP significantly reduced the particle size before heating from 430 nm in the control to 234 nm at 1 min of treatment. This reduction indicated disruption of pre-existing EWP-CN aggregates through plasma-induced etching, partial unfolding, and cleavage of weakly associated protein structures. Similar aggregate dissociation and exposure of polar groups were observed in ACP-treated sunflower and soybean proteins [21,22]. Beyond 1 min, particle size gradually increased, suggesting that oxidation-induced protein reassociation began to compete with plasma-mediated disaggregation. Meanwhile, heating increased particle size in each treatment, but the extent of this increase depended strongly on ACP exposure. The sample treated for 1 min retained the smallest heated particles (290 nm), confirming its superior resistance to thermal aggregation. In contrast, particle size increased remarkably to 522 nm at 3 min and 1103 nm at 5 min (*p* < 0.05). Controlled plasma treatment partially unfolded proteins and exposed charged regions that enhance hydration. However, prolonged exposure intensified protein reassociation, resulting in larger and more heterogeneous particles during subsequent heating [23]. Likewise, Tang et al. [24] showed that short plasma treatment modified ovalbumin through controlled polypeptide cleavage, whereas extensive structural modification increased its susceptibility to reassociation.

As shown in Figure 2B, the PDI results further distinguish between particle size and the homogeneity of its distribution. Before heating, no significant differences were observed among treatments (*p* > 0.05). After heating, the control and 1 min samples displayed the lowest PDI values among all treatments. Extended ACP generated increasingly heterogeneous aggregates containing both small and large particles. The high PDI values indicate very broad particle distributions, limiting the quantitative reliability of the DLS-derived diameters. Thus, these values are interpreted cautiously as qualitative indicators of sample heterogeneity and aggregation rather than precise particle sizes. This behavior is clearly reflected in the size distribution profiles (Figure 2C,D). Before heating, all dispersions showed broad or multimodal distributions, although ACP shifted part of the particles toward smaller sizes. After heating, the treatment for 1 min maintained a relatively broad but smaller particles, while the treatments for 3 and 5 min markedly shifted the particle sizes toward large clusters. The shift toward large particle clusters after 5 min indicated extensive aggregation and possible network development but did not establish specific crosslinks. Similarly, plasma-assisted ovalbumin amyloid fibrils have been attributed to protein unfolding followed by hydrophobic association and dityrosine crosslinking [25]. Additionally, electrophoretic mobility remained approximately −2.0 μmcm/Vs before heating and became more negative (−2.5 to −2.7 μmcm/Vs) after heating, with no significant effect of ACP duration within either condition (Figure 2E).

In parallel, heating increased the ζ-potential from –28 mV to –36 mV after 1 min of treatment (Figure 2G), indicating exposure of negatively charged acidic residues. This suggested that enhanced electrostatic repulsion contributed to the thermal stability of the 1 min treatment-induced effects. Wang et al. [21] associated increased negative ζ-potential after plasma treatment with exposure of polar and ionizable protein groups. However, the more negative ζ-potential at longer treatments did not prevent aggregation, demonstrating that surface charge alone could not overcome strengthened hydrophobic and covalent interactions. Furthermore, electrical conductivity was relatively stable before heating but generally decreased after heating for up to 3 min, possibly because ions became associated with or entrapped within the protein aggregates (Figure 2F). In contrast, conductivity increased sharply to 0.21 mS/cm after 5 min of treatment. This might reflect the accumulation of plasma-generated ionic species and the exposure or release of ionizable groups during severe oxidation and heating. Increased ionic strength can compress the electrical double layer and screen electrostatic repulsion, even when the measured ζ-potential remains relatively negative. Consequently, protein particles can approach sufficiently closely for hydrophobic association, disulfide exchange, and oxidative crosslinking. These results were consistent with those of Nasiru et al. [13] for ACP-treated EWPs.

### 3.3. UV Absorption Analysis

**Figure 3:**
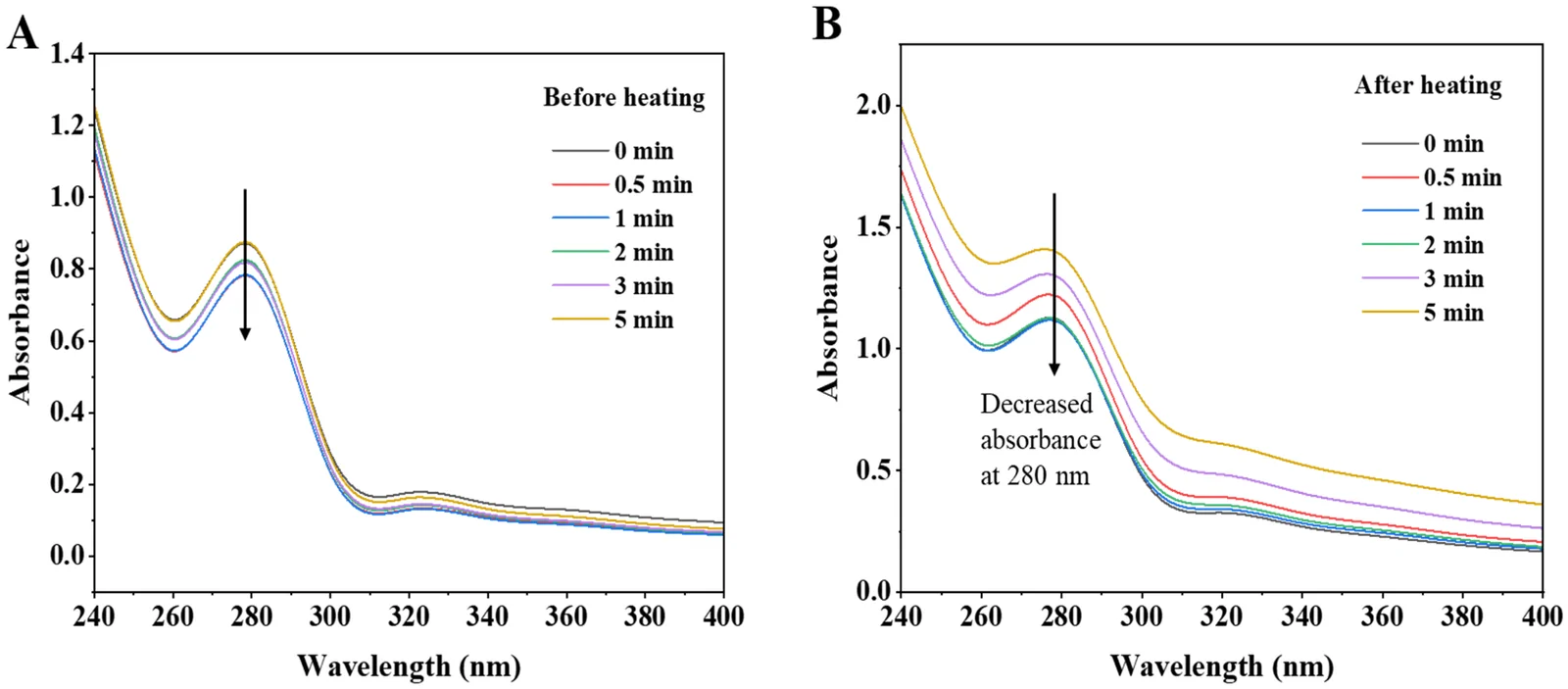
UV spectra before (**A**) and after heating (**B**) of ACP-treated EWP-CN dual complexes at different times (min).

UV absorption near 280 nm reflects the microenvironment of aromatic residues and is sensitive to changes in protein tertiary structure. Before heating, all treatments retained a maximum near 280 nm, indicating no major shift in the aromatic chromophore environment (Figure 3A). Nonetheless, the treatments for 1 and 2 min slightly reduced absorbance, whereas the 3 and 5 min treatments increased it, particularly the 5 min treatment. This suggested that mild ACP disrupted loosely associated particles and reorganized aromatic residues, consistent with the reduced particle size and improved solubility observed. On the contrary, prolonged exposure promoted unfolding and greater exposure of tyrosine (Tyr), tryptophan (Trp), and phenylalanine (Phe) residues, similar to spectral changes reported for plasma-treated EWPs and pea proteins [26,27]. Heating markedly amplified these differences, with the treatments for 3 and 5 min showing the highest absorbance and pronounced tails between 300 and 400 nm (Figure 3B). Because protein chromophores absorb weakly in this region, the elevated baseline mainly indicated light scattering from large aggregates. This agreed with the increased particle size, PDI, turbidity, and reduced solubility of these treatments. In contrast, the lower absorbance of treatments for 1 and 2 min corresponded to their smaller particles and greater colloidal stability. Mechanistically, moderate ACP caused limited unfolding that facilitated controlled EWP-CN association, whereas prolonged treatment might intensify oxidation, hydrophobic interactions, sulfhydryl–disulfide exchange, and covalent crosslinking. Subsequent heating accelerated these interactions, producing extensive aggregation and gelation at 5 min. Comparable plasma-induced cleavage and dityrosine-mediated association have been reported during heat-induced ovalbumin assembly [25], while severe plasma conditions promoted oxidation and aggregation in whey protein isolates [23]. Thus, the UV spectra confirmed a transition from beneficial structural rearrangement at 1–2 min to overprocessing and network formation at 5 min.

### 3.4. Intrinsic Fluorescence Emission Analysis

**Figure 4:**
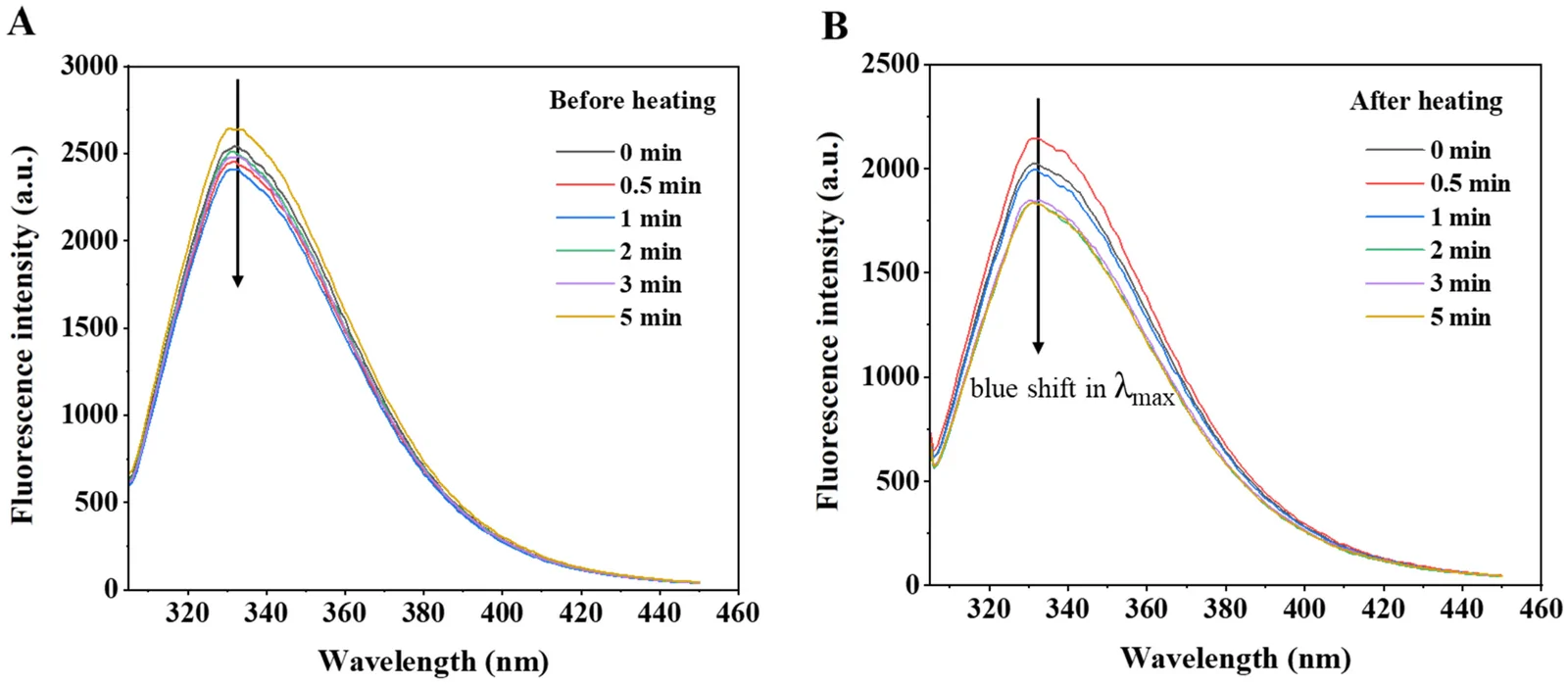
Intrinsic fluorescence spectra before (**A**) and after heating (**B**) of ACP-treated EWP-CN dual complexes at different times (min).

Intrinsic fluorescence primarily reflects the microenvironment of Trp residues and is sensitive to changes in tertiary structure [9]. As shown in Figure 4A, all samples before heating showed a maximum near 334 nm, with only modest intensity differences. The 1 min treatment produced the lowest intensity, suggesting limited unfolding and increased solvent exposure. The higher intensity at 5 min indicated reassociation and partial burial of aromatic residues within ACP-induced aggregates. Similar dose-dependent conformational rearrangements have been reported for plasma-treated sunflower and soybean proteins [21,22]. Additionally, heating reduced the overall fluorescence intensity and caused a slight blue shift in the maximum emission wavelength (λ_max_) (Figure 4B). The treatment for 0.5 min retained the highest intensity, while longer treatments, particularly 3 and 5 min, showed stronger fluorescence quenching. The simultaneous blue shift and intensity loss suggested that heating transferred Trp residues into less polar aggregated domains, while oxidation and close protein-protein contacts promoted static quenching. Waqar et al. [28] associated decreased Trp fluorescence in plasma-activated EWPs with aggregation and tertiary structure disruption. Moreover, plasma-induced hydrophobic association and dityrosine crosslinking can strengthen heat-driven ovalbumin assembly [25]. When considered with the particle size results, the quenching at 1 and 2 min reflects controlled EWP-CN association, while the low intensity at 5 min coincided with high turbidity, large heterogeneous particles, reduced solubility, and heat-induced gelation, collectively indicating extensive oxidation and aggregation.

### 3.5. Surface Hydrophobicity Analysis

**Figure 5:**
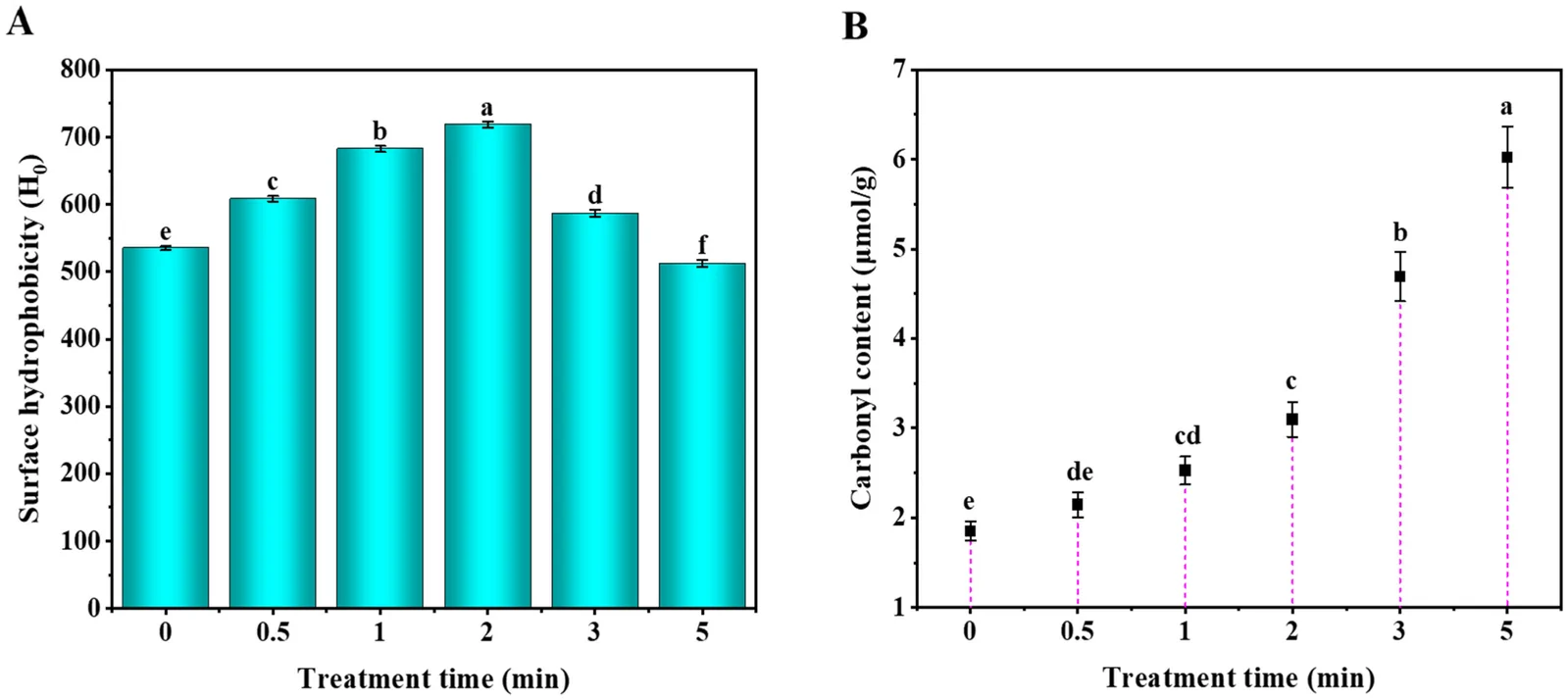
Surface hydrophobicity (**A**) and carbonyl content (**B**) of ACP-treated EWP-CN dual complexes at different times. Different letters represent significant differences at *p* < 0.05.

The surface hydrophobicity (H_0_) property was measured prior to heating, and thus mainly reflected the structural changes resulting from ACP. As depicted in Figure 5A, the H_0_ increased progressively from 535.16 ± 2.95 in the control to 718.60 ± 4.15 at 2 min of ACP treatment (*p* < 0.05), corresponding to a 34.3% increase. This might be due to the generated RONS that partially unfolded EWPs and rearranged complex interactions, exposing previously buried nonpolar residues to ANS. Casein’s flexible structure may further promote association with these exposed hydrophobic regions. Similar ACP-induced structural rearrangement and functional improvement have been reported for EWPs and ovalbumin [13,24].

In addition, H_0_ declined sharply (*p* < 0.05) at 3 and 5 min to 586.86 ± 4.65 and 512.31 ± 4.74, respectively. The decline in H_0_ after 3–5 min coincided with greater carbonyl accumulation and aggregation, suggesting burial of hydrophobic regions and restricted ANS accessibility. This was consistent with the findings of Baek et al. [14], who noted increased H_0_ for EWPs during moderate ACP treatment, followed by a decrease under excessive exposure because of aggregation. The decline at 3 and 5 min also corresponded with the increased particle size, PDI, turbidity, reduced solubility, and eventual heat-induced gelation observed in these systems. Waqar et al. [28] similarly associated plasma-induced changes in EWP hydrophobicity with decreased sulfhydryl content, increased dityrosine formation, tertiary structure disruption, higher turbidity, and aggregation. Dai et al. [29] demonstrated that plasma exposure significantly altered the H_0_ and structural organization of desalted duck EWPs. Overall, ACP initially promoted controlled protein unfolding and hydrophobic region exposure during 0.5 to 2 min. Longer treatment (3 and 5 min) intensified oxidative crosslinking and aggregation, burying these regions within the complexes. Thus, 2 min produced the highest H_0_ under the tested conditions, but did not represent an the optimum time across all properties.

### 3.6. Protein Oxidation Analysis

Protein oxidation is a central mediator of ACP-induced structural modification because plasma-generated reactive species attack susceptible amino acid residues, while carbonyl formation provides a stable index of the accumulated oxidative load [11]. As shown in Figure 5B, the carbonyl content increased progressively from 1.85 ± 0.10 μmol/g in the control to 2.14 ± 0.13, 2.53 ± 0.15, and 3.09 ± 0.19 μmol/g after 0.5, 1, and 2 min, respectively. However, adjacent treatments within this range were not consistently different, as indicated by their shared letter groups. More pronounced and significant increases occurred at 3 and 5 min, reaching 4.69 ± 0.27 and 6.02 ± 0.34 μmol/g, respectively (*p* < 0.05). Concurrently, carbonyl content increased non-linearly with ACP exposure. The sharpest increase occurred between 2 and 3 min, followed by a slower increase from 3 to 5 min, indicating treatment-dependent accumulation rather than continuously accelerating oxidation. RONS commonly reported in ACP systems, including •OH, O_3_, and H_2_O_2_, may oxidize susceptible amino acid residues and potentially promote disulfide or dityrosine crosslinking; however, these species and crosslinks were not directly measured in this study. Likewise, Zhang et al. [15] reported increased carbonyl content and decreased free sulfhydryl groups in cold plasma-treated CN, confirming its susceptibility to plasma oxidation. The moderate carbonyl accumulation up to 2 min was associated with partial unfolding and exposure of previously buried hydrophobic regions, consistent with the increase in H_0_. Marked carbonyl accumulation at 3 and 5 min indicated greater oxidation and coincided with aggregation. The decline in H_0_ suggested that ANS-accessible hydrophobic regions became buried within aggregates; however, the responsible intermolecular interactions were not directly determined. Visible heat-induced gelation occurred after 3 and 5 min of plasma treatment. This time-dependent transition agrees with the results of Baek et al. [14], who noted increased EWP unfolding and H_0_ under moderate ACP treatment but aggregation and reduced hydrophobic exposure after excessive treatment. Additionally, reactive oxygen species-driven oxidation has been shown to regulate EWP conformation and intermolecular interactions [30]. Accordingly, the carbonyl and hydrophobicity results collectively identified 1–2 min as the controlled structural modification stage, whereas 3 and 5 min induced progressive oxidation, aggregation, and network formation.

### 3.7. Emulsifying and Foaming Properties Analysis

**Figure 6:**
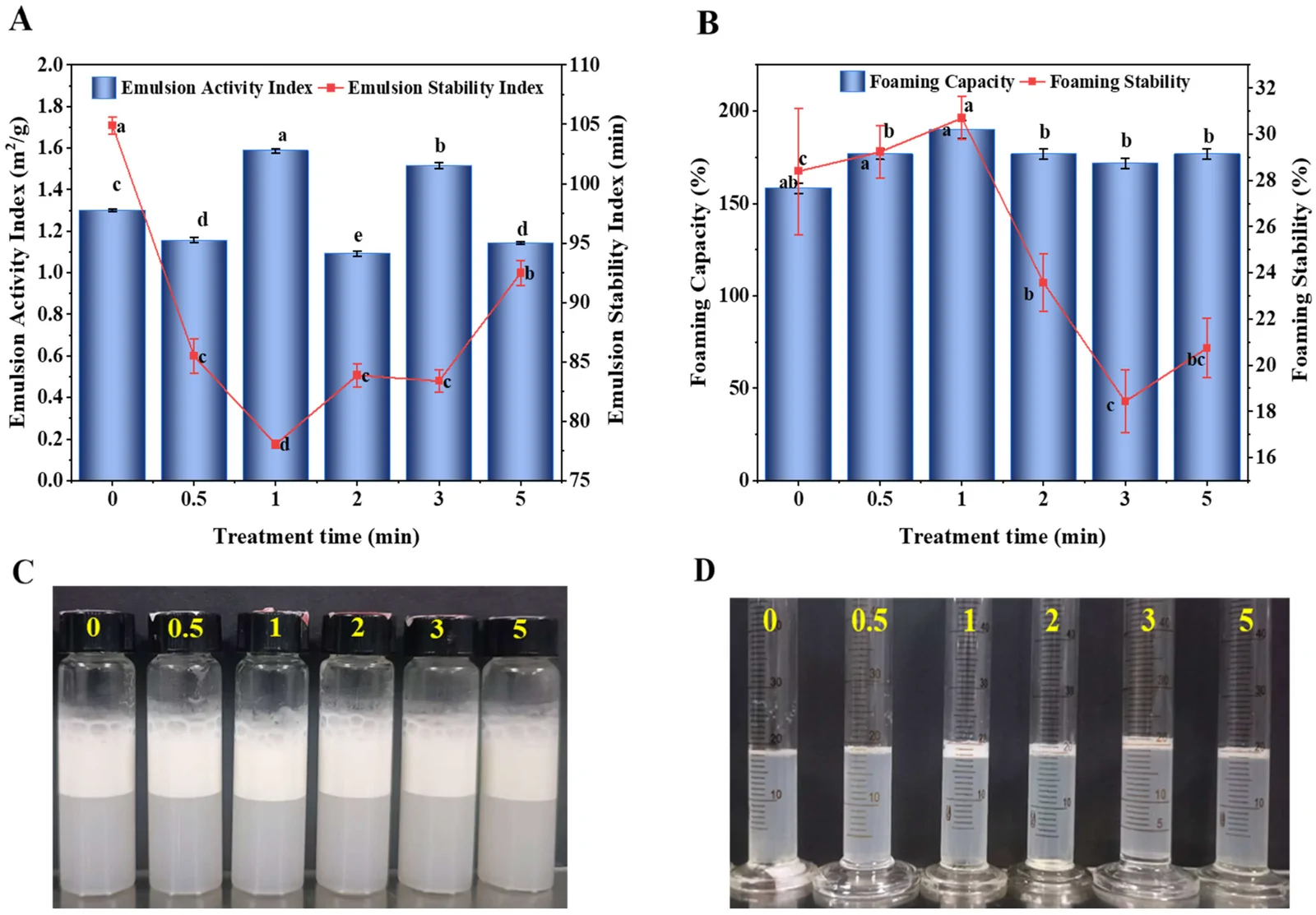
Emulsifying activity index and emulsion stability index (**A**), and foaming capacity and foaming stability (**B**) of ACP-treated EWP-CN dual complexes at different times. Different lowercase letters indicate significant differences among samples for the same parameter (*p* < 0.05). Visual appearance of the emulsions (**C**) and foams (**D**) after standing for 24 h.

Emulsifying and foaming properties reflect the ability of proteins to adsorb rapidly and form cohesive films at oil–water and air–water interfaces. ACP treatment produced a non-linear effect on EWP-CN system emulsification (Figure 6A). The EAI increased from 1.30 m^2^/g in the control to a maximum of 1.59 m^2^/g at 1 min, decreased at 2 min, and increased again to 1.51 m^2^/g at 3 min. The improvement at 1 min aligns with its smaller particle size and higher solubility, which facilitated protein diffusion and adsorption at the oil-water interface. Limited unfolding may also expose hydrophobic regions while retaining sufficient molecular flexibility for interfacial rearrangement. Nevertheless, H_0_ alone did not explain the EAI trend. Although it peaked at 2 min, EAI fell below the control, suggesting that aggregation or restricted interfacial rearrangement offset the benefit of hydrophobic exposure. Conversely, the recovery of EAI at 3 min despite lower hydrophobicity indicates that particle size, solubility, aggregation state, and molecular flexibility collectively governed emulsifying activity. Similar results for plasma-induced improvements in ovalbumin emulsification were observed in previous studies [20,24]. Wang et al. [31] reported increases of 102% and 63% in the emulsifying and foaming capacities of plasma-modified peanut protein, respectively. Despite the increased EAI at 1 min, ESI decreased from 105 min in the control to 78 min and remained below the control in all ACP-treated samples. Thus, ACP enhanced initial emulsifying activity at 1 min but did not improve emulsion stability. The greater interfacial area generated by highly active proteins may have resulted in incomplete droplet coverage and subsequent coalescence. After 5 min of treatment, ESI partially recovered despite the lower EAI, probably because larger crosslinked aggregates formed thicker and less-mobile interfacial layers. This was in agreement with the findings of Liu et al. [20], who demonstrated that controlled plasma modification of ovalbumin enhanced emulsification by limiting thermal aggregation. Li et al. [32] found that moderate cold plasma treatment unfolded rice bran protein and improved the stability of vegetable oil-based whipped cream, while excessive structural modification was unfavorable. However, the appearance of a visible creamy layer after 24 h (Figure 6C) confirmed that none of the complex systems completely prevented gravitational separation.

As illustrated in Figure 6B, foaming capacity and stability were both highest at 1 min, reaching approximately 190 and 31%, respectively. This treatment provided an appropriate balance among solubility, molecular flexibility, surface hydrophobicity, and particle size, enabling rapid adsorption and formation of cohesive air–water films. Krichanã et al. [33] showed that intermediate plasma intensity improved the interfacial and rheological properties of aquafaba and generated highly stable aerated systems. Conversely, longer ACP treatments maintained relatively high FC but sharply reduced FS, with the lowest value observed at 3 min. Excessive oxidation, aggregation, and crosslinking likely restricted molecular diffusion and interfacial rearrangement, producing heterogeneous films susceptible to drainage and bubble coalescence. The extensive foam collapse after 24 h (Figure 6D) supported this mechanism. Overall, ACP treatment for 1 min enhanced initial interfacial performance, yielding a higher EAI, foaming capacity, and foam stability than the control. Nevertheless, ESI remained lower than the control across all treatments, and visible creaming after 24 h confirmed that long-term emulsion stability was not improved. At 2 min, both EAI and foam stability fell below those of the control, whereas prolonged treatment (3–5 min) promoted aggregation and further weakened interfacial stability.

## 4. Proposed Mechanism for ACP-Assisted EWP-CN Dual Complexation

Before ACP treatment, the EWP-CN system likely existed as loosely associated colloidal complexes. At pH 7.0, both protein fractions carry predominantly net negative charges. Therefore, the complexation was mainly governed by hydrophobic interactions, hydrogen bonding, van der Waals forces, and localized electrostatic contacts rather than bulk electrostatic attraction. The flexible CN chains associated with hydrophobic patches on globular EWPs provided steric and electrostatic stabilization against uncontrolled aggregation, resulting in stable EWP-CN colloidal complexes [5]. Salama et al. [8] identified hydrophobic interactions as a major mechanism underlying their improved thermal stability. At 1–2 min, RONS generated by ACP might induce moderate oxidation and partial EWP unfolding. This interpretation was supported by the increase in H_0_ from 535.16 in the 0 min control to 718.60 after 2 min of treatment, as well as the moderate increase in carbonyl content from 1.85 to 3.09 μmol carbonyl/g protein. The accompanying UV, intrinsic fluorescence, and particle size changes further indicated rearrangement of the protein tertiary structure. In addition, exposure of previously buried nonpolar regions strengthened hydrophobic associations between EWPs and the flexible CN chains without producing extensive crosslinking. CN may have influenced the association of partially unfolded EWP molecules; however, a specific shielding effect cannot be established from the present measurements. Also, the residual surface charge and the extended CN chains provided additional electrostatic and steric stabilization. This protective organization explains the relatively high solubility and absence of gelation after heating at 90 °C. Liu et al. [20] showed that controlled plasma oxidation of hydrophobic and sulfhydryl sites inhibited heat-induced ovalbumin aggregation, while Baek et al. [14] associated moderate ACP treatment with EWP unfolding and increased H_0_.

**Figure 7:**
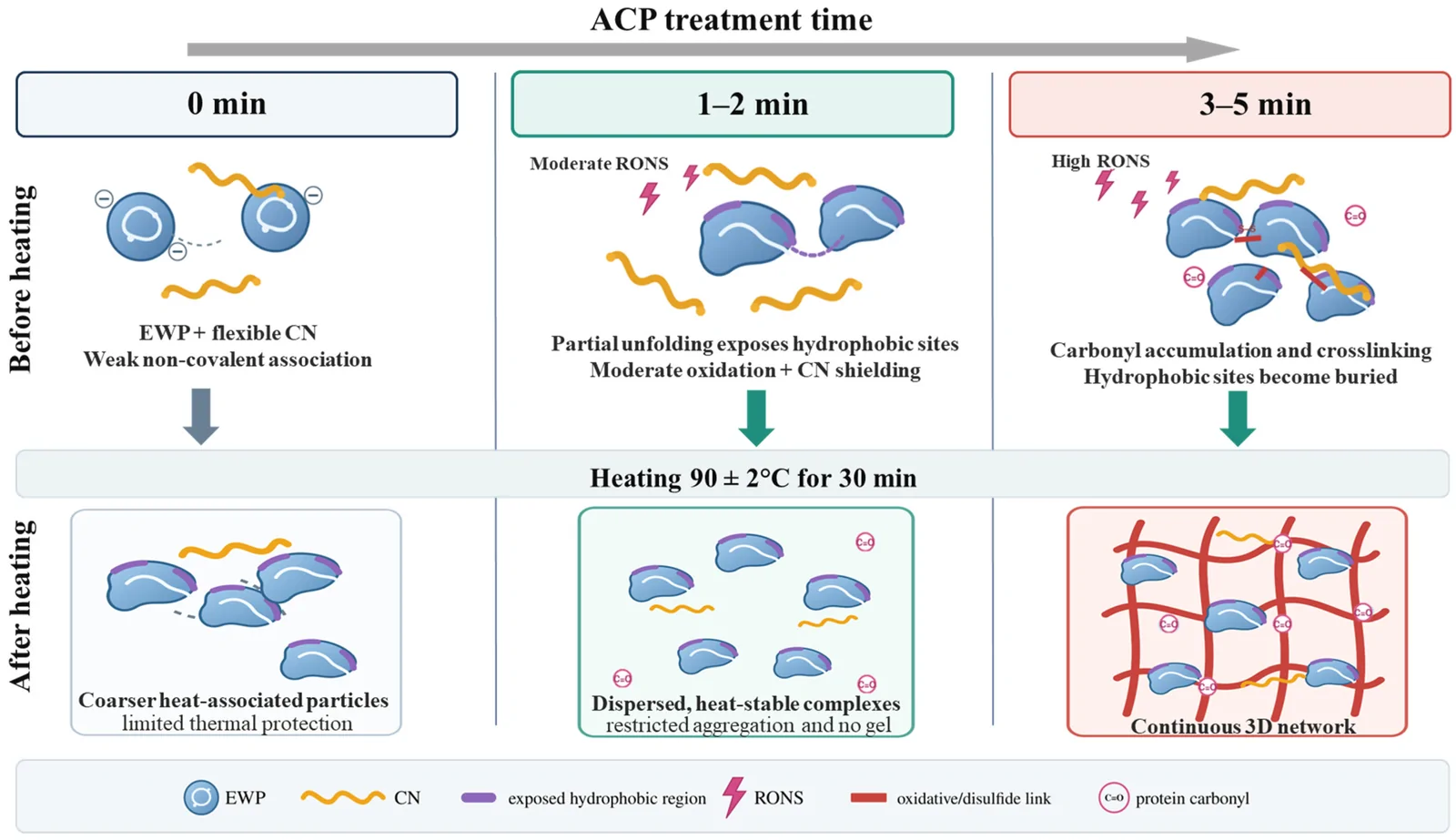
Proposed mechanism of ACP-induced structural changes in EWP-CN dual system.

In contrast, treatment for 3–5 min exceeded the controlled modification range. The carbonyl content increased sharply to 6.02 μmol/g at 5 min, whereas H_0_ decreased to 512.31. This decline does not indicate protein refolding. Indeed, the increased turbidity and particle size, and reduced solubility suggest that hydrophobic regions became buried within newly formed aggregates. Prolonged exposure to plasma-derived radicals likely intensified oxidation of sulfhydryl, methionine, aromatic, and other susceptible residues, potentially promoting disulfide formation, hydrophobic association, and other covalent or non-covalent crosslinks. Zhang et al. [15] observed carbonyl accumulation and sulfhydryl loss in plasma-treated CN, confirming that casein can also participate in oxidation-driven association. During subsequent heating, ACP-modified EWPs and CN might undergo further unfolding, with hydrophobic associations, hydrogen bonding, sulfhydryl–disulfide exchange, and covalent crosslinking potentially contributing to network formation. At this stage, CN may have participated in aggregate formation, although its proposed bridging role remains to be confirmed experimentally. Once the concentration and connectivity of these aggregates exceeded the network formation threshold, they became junction zones within a continuous 3D structure that immobilized water, producing gelation at 3–5 min. Thus, ACP produced two distinct regimes: controlled oxidation and thermally stable dual complexation at 1–2 min, followed by excessive oxidation, co-aggregation, and heat-induced network formation at 3–5 min (Figure 7). This proposed mechanism agrees with the established transition from beneficial protein unfolding to irreversible aggregation under excessive plasma exposure [11,31]. The results confirm ACP-induced changes in aggregation, surface charge, conformation, hydrophobicity, and oxidation within the mixed protein system. However, they neither distinguish EWP-EWP, CN-CN, and EWP-CN associations nor directly confirm CN-mediated shielding, bridging, or specific crosslinks. These mechanisms therefore remain literature-supported interpretations requiring protein-specific analyses.

## 5. Conclusions

This study highlighted that ACP produced treatment-dependent changes in the structure, colloidal behavior, and functionality of the mixed EWP-CN system. Treatment for 1 min produced the smallest particles and increased EAI, foaming capacity, and foam stability relative to the control. After 2 min of plasma treatment, surface hydrophobicity reached its maximum, although EAI and foam stability remained below those of the control. Treatment for 3–5 min resulted in greater oxidation and aggregation, while subsequent heating produced a visible gel after 5 min, demonstrating a transition from controlled complexation to extensive network formation. Thus, under the tested conditions, 1–2 min represented a property-dependent modification window rather than a uniform improvement in functionality. This work provides a basis for designing EWP-CN dual ingredients with adjustable colloidal stability, thermal behavior, and functionality. Further studies should investigate different protein ratios, concentrations, and ACP voltages and validate process scalability, storage stability, and performance in real food matrices.

## Data Availability

Data are available upon request from the corresponding author.
